# Professional assessment compared to patients’ attitudes toward tooth replacement: a cross-sectional study

**DOI:** 10.1186/s12903-023-03355-7

**Published:** 2023-09-05

**Authors:** Haidar Alalawi, Hasan Alhumaily

**Affiliations:** 1 Department of Substitutive Dental Sciences, College of Dentistry, Imam Abdulrahman Bin Faisal University, P. O. Box 1982, Dammam, 31441 Saudi Arabia; 2 Consultant, Prosthodontic Department, AlAhsa Dental Center, Ministry of Health, AlAhsa, Saudi Arabia

**Keywords:** Knowledge, Attitudes, Missing, Teeth replacement, Dental, Saudi Arabia

## Abstract

**Background:**

There is a difference between patient self-assessment and professional assessment of oral health needs; therefore, the aim of the study was to investigate patients’ individual needs and awareness of replacing missing teeth with prostheses and then to compare this information with professionally assessed clinical prosthetic needs in the Eastern Province of Saudi Arabia.

**Methods:**

This was a cross-sectional study conducted in the Eastern Province of Saudi Arabia. The study subjects were recruited from Imam Abdulrahman bin Faisal University in Dammam City, Primary Health Care Centers in Alhasa City and from health education campaigns in the same area. All the patients were provided with a questionnaire related to the effect of missing teeth and replacement options, then underwent a clinical examination performed by a well-trained investigator. Statistical analyses were performed using JMP data analysis software (JMP®, Version 16. SAS Institute Inc., Cary, NC, 1989–2021.)

**Results:**

A total of 102 participants were included. Most of the participants (94.2%) reported their need to replace missing teeth. Most of the participants stated that losing teeth (teeth) affected their ability to chew food and their appearance (82.6% and 61.6%, respectively). Dental caries was the main reason behind teeth extraction in 77.9% of the study sample. Fixed partial prosthesis was the first treatment option preferred by 33.7%, followed by implant-supported prosthesis with 25.6% to replace the missing teeth. Only 3.5% of participants preferred not to restore the missing teeth. Professional screening showed that 48.8% of the participants had one missing anterior tooth or more, which dictates the need for esthetic restoration, and 58.1% of the participants had three missing posterior teeth or more, which dictates the need for functional restoration.

**Conclusions:**

Patient knowledge and attitudes toward replacing missing teeth in terms of their functional and esthetic needs were variable among the population in comparison to the professional assessment of patient needs. Dentists plays a major role in raising the level of awareness about missing teeth replacement. The results of this study serve as baseline data for any related future studies.

## Background

Teeth play a major role in reflecting personality and attitude regarding self-image. Losing teeth reduces a person’s quality of life psychologically, socially, and emotionally. Currently, dentistry correlates the emotions and psychology of patients in relation to dental situations, especially esthetics [1]. Losing teeth is very traumatic and can disturb social activity such that a requires significant psychological treatment [2, 3].

Replacement of missing teeth to restore function and esthetics had different modalities of treatment, including dental implants, fixed partial dentures, and removable partial dentures. Each modality has its own advantages and disadvantages [4]. The final treatment decision is affected by several factors, and it is case-dependent. If more than one option is possible, the definitive prosthesis depends on the patient’s decision. It is recommended to assess knowledge and attitude toward prostheses just to ensure patient satisfaction [5, 6]. Therefore, the final decision of treatment cannot depend on the opinion of the dentist alone but should be discussed closely with the patient [7].

Assessment of a prosthodontic patient’s needs is based on the location and length of edentulous space [8, 9]. Researchers [9] reported that social and esthetic reasons were the basis for restoring missing teeth, and the decision to restore missing teeth did not only rely on professional assessment. Several studies have stated the presence of disagreement between dentists’ and patients’ assessments [8, 10, 11].

The level of awareness and perceptions among patients toward dental restorations and replacements vary in different cultures and populations. A study performed in the Kingdom of Saudi Arabia reported that subjective perceptions of esthetic and functional treatment needs were highly variable among male patients [12]. Another survey conducted in Hyderabad India reported that the patient’s awareness of diverse treatment options for missing teeth was low [13]. Additionally, a study conducted on the Chinese adult population found that 62% of the subjects had no tooth replacement, 30% had teeth replaced by FDP, and 11% had teeth replaced by RDP. 3% of the subjects had both FDP and RDP [14] In Europe, the frequency of removable dentures varied between 13 and 29%, with 3–13% of individuals wearing upper and lower complete dentures [15]. In Sudan, a study reported that 57% of subjects were in need of prosthetic replacement, which may reflect a lack of access to dental services and possibly a lack of dental awareness among the population [16].

The objective of this study was to report the perceived prosthetic treatment needs of a sample of patients and the factors that influenced their perceptions, as well as to compare these perceived needs to professionally evaluated clinical needs.

## Methods

### Study design

This was a cross-sectional study. This study was conducted through self-administered surveys and clinical assessments.

### Study subjects

A pilot study with thirty participants was carried out at Imam Abdulrahman bin Faisal University’s College of Dentistry. Based on this, the sample size was determined at 99% confidence level with a 5% margin of error. Based on power analysis, a minimum sample size of 76 participants was determined. The subjects were included based on the inclusion criteria of being Saudi, completing the survey and receiving a clinical oral examination; all non-Saudi individuals were excluded. The subjects were divided into 4 age groups: 18–25, 26–35, 36–45, and < 45 years. The study sample was recruited from Imam Abdulrahman bin Faisal University College of Dentistry in Dammam City, primary health care centers in Alhasa City, and health education campaigns in the same area.

### Data Collection

#### Self-administered survey

In the self-administered survey provided in English and Arabic, the following information was obtained from October until December 2022: age, gender, level of education, occupation, economic status, marital status, nationality, systemic diseases, the need to replace a missing tooth/teeth, the effect of losing teeth on appearance, the effect of losing teeth on chewing ability, causes of tooth/teeth loss, preferred treatment option, and factors that create hindrances for treatment.

### Clinical Assessment

Professional assessment of patients’ needs was performed by a trained dentist through examination sheets. The examination sheet reflected the following information: the number of missing anterior or posterior teeth. The need for functional or esthetic restoration was assessed according to the following criteria: at least 1 missing anterior tooth (incisor, canine, or premolar) constituted a need for esthetic restoration and at least 3 missing posterior teeth (premolar, first molar, second molar) constituted a need for functional restoration. Additionally, the provided prosthesis type was recorded if a missing tooth was restored.

If subjects did not perceive a need for treatment but professional assessment indicated otherwise (positive need), the outcome was categorized as underestimation. If subjects perceived a need for treatment but professional assessment indicated otherwise (negative need), the outcome was categorized as overestimation. Probabilities of overestimation for each age group and educational level were calculated with the following formula:

[No. of subjects (overestimations)/Total no. of subjects (negative professionally assessed need)] x 100.

Similarly, the probabilities of underestimation for each age group and educational level were calculated as follows:

[No. of subjects (underestimations)/Total no. of subjects (positive professionally assessed need)] x 100.

Statistical analyses were performed using JMP data analysis software (JMP®, Version 16. SAS Institute Inc., Cary, NC, 1989–2021), and a P value of less than 0.05 was considered to indicate statistical significance. Data were subjected to the calculation of frequency distributions. The chi-square test was used, and numerical variables are described as the mean and standard deviation. The perceived need was compared to professionally assessed needs according to the 4 age groups and 3 educational levels with Bowker’s Test (equivalent to McNemar’s Test) at the 5% level of significance. The study was approved by the Ethics Committee of the College of Dentistry, Imam Abdulrahman bin Faisal University.

## Results

**Fig. 1:**
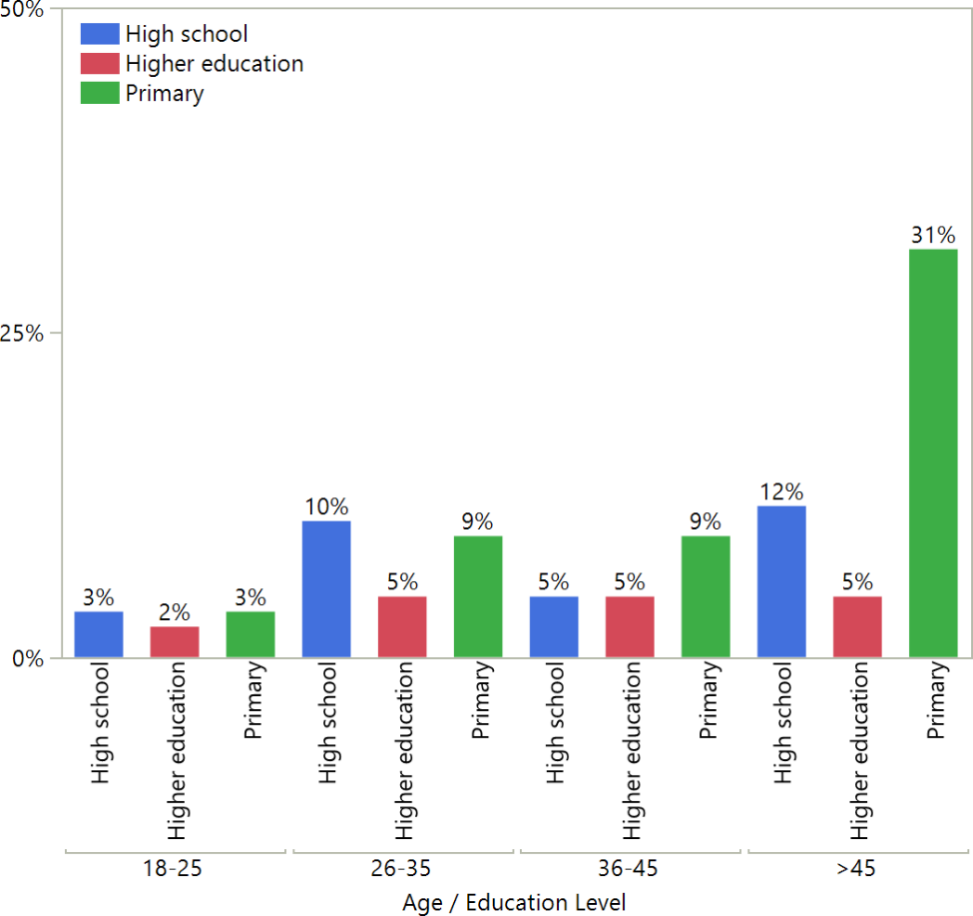
Distribution of participant age and educational level

**Fig. 2:**
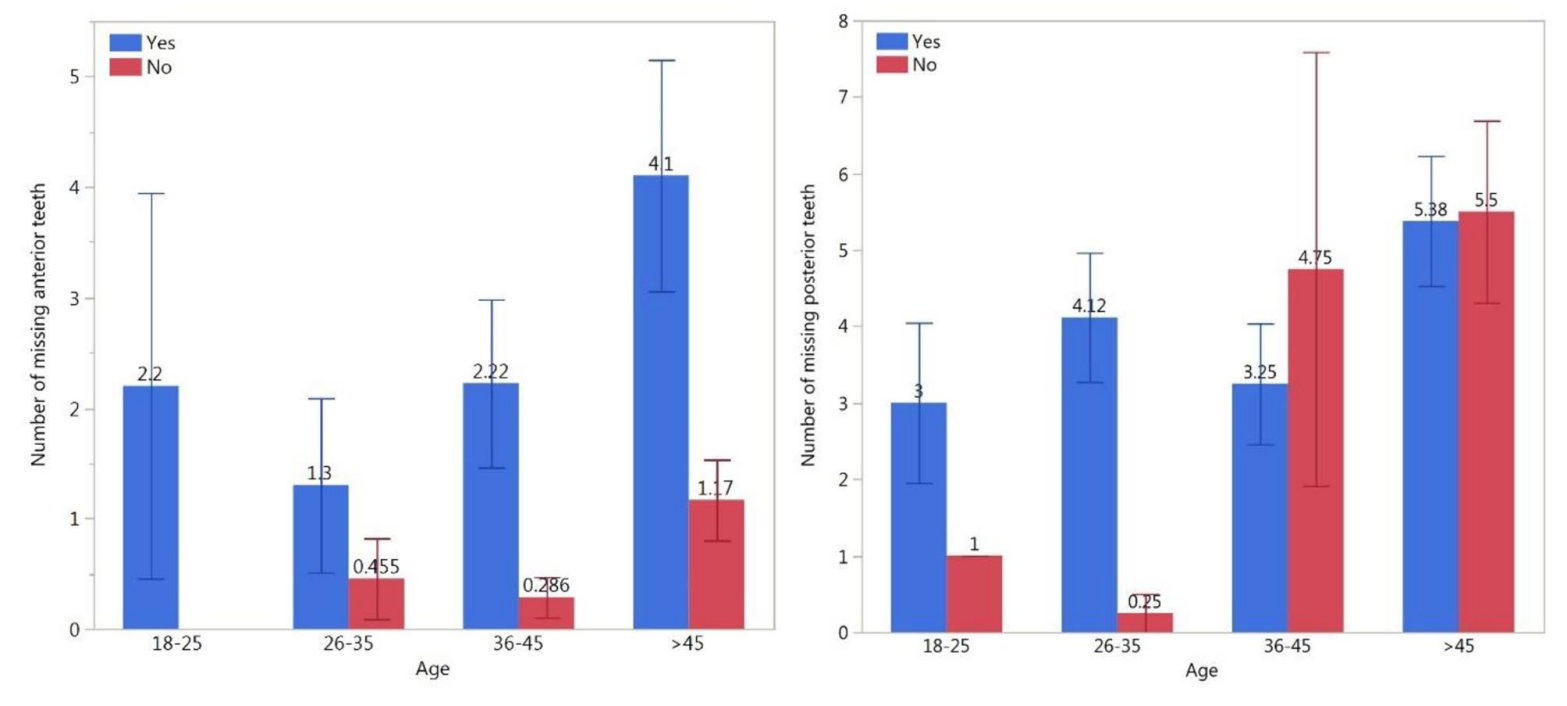
Mean and standard error of missing anterior and posterior teeth according to responses to Q9 categorized by age group

The total sample size was 102 participants, with 47 males (46.1%) and 55 females (53.9%). The mean age of the study sample was 42.26 (± 14.98) years, ranging from 16 to 77 years. The distribution of the subject’s age and education level is shown in Fig. 1. A few participants had systemic diseases, such as hypertension (15.7%), diabetes (20.6%), and other chronic diseases (13.7%). The mean number of missing anterior teeth per subject was 2.14 ± 3.88, and the mean number of missing posterior teeth per subject was 4.28 ± 4.26. A higher number of missing anterior and posterior teeth was associated with an increase in age in participants with a perceived need for tooth replacement (Fig. 2).

**Fig. 3:**
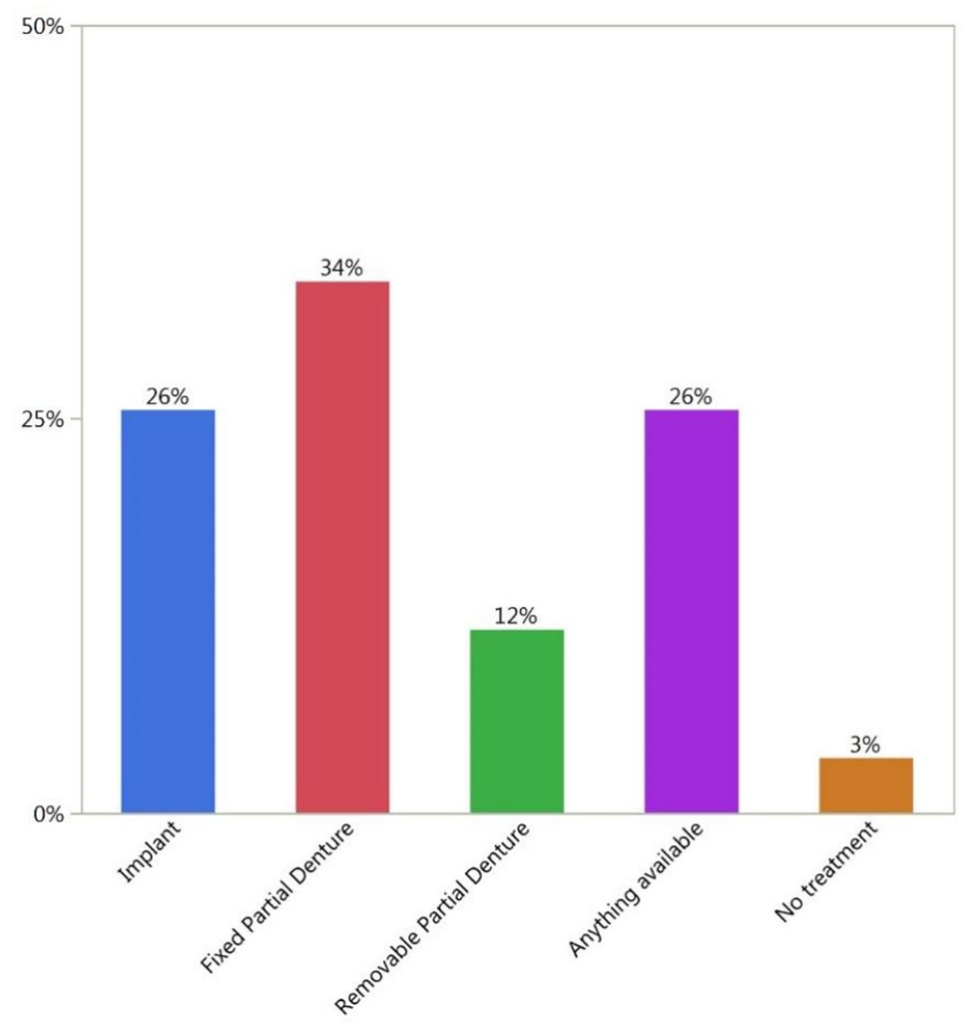
Preferred treatment options by all participants

The distribution of the preferred treatment option is shown in Fig. 3. The distribution of the level of education and preferred treatment options is shown in Table 1. Table 2 shows participants’ perspectives on the impact of missing teeth on cosmetic and functional aspects.

**Table 1:** Relationship between the level of education and preferred treatment options

Education level	Implant	Fixed partial denture	Removable partial denture	Anything available	No treatment
Primary	10.47%	19.77%	8.14%	13.95%	1.16%
High school	11.63%	6.98%	2.33%	8.14%	1.16%
Higher education	3.49%	6.98%	1.16%	3.49%	1.16%

**Table 2:** Distribution of responses to 3 yes/no questions

Questions	Yes: number (%)	No: number (%)
Do you think you need to replace the missing tooth (teeth)?*	81 (94.19)	5 (5.81)
Do you think losing your tooth (teeth) has affected your appearance?	53 (61.63)	33 (38.37)
Do you think losing your tooth (teeth) has affected your ability to chew food?	71 (82.56)	15(17.44)

**Fig. 4:**
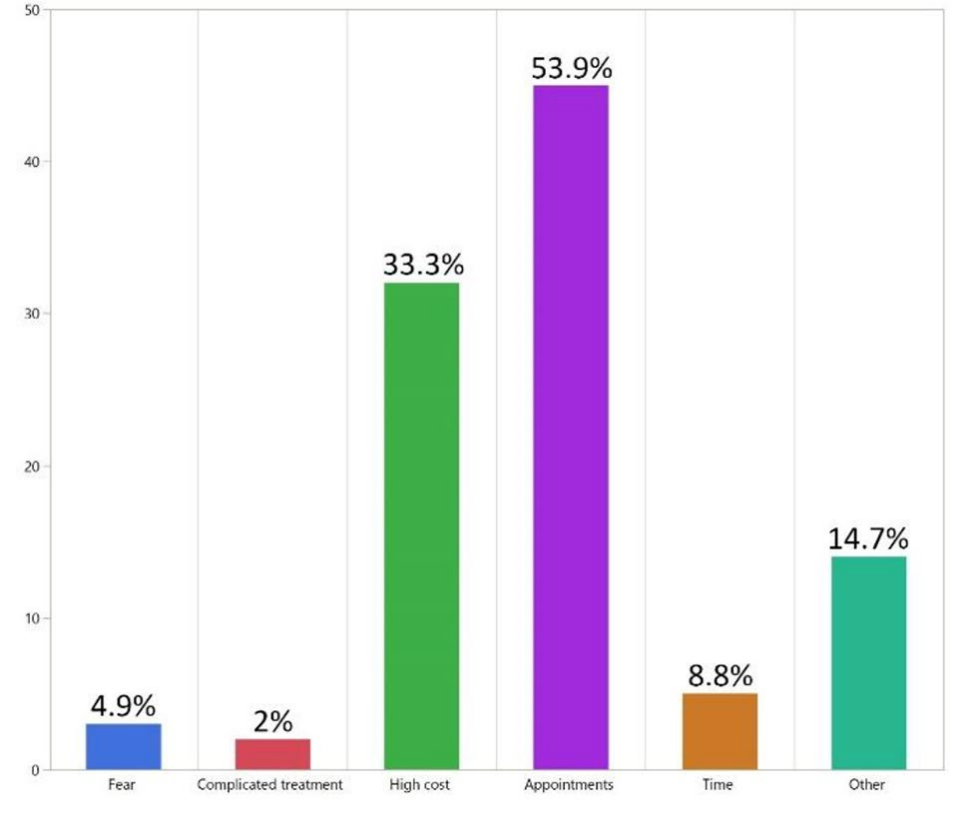
Reasons missing teeth were not replaced

Regarding the source of knowledge about the possible treatment options, most of the participants had been informed about the treatment options by a dentist (61.8%), while 27.5% of the participants had been informed about the treatment options by a friend. The most common reason for tooth loss was dental caries, as was the case in 79.4% of the participants, followed by periodontal disease in 18.6% of the participants, and then trauma in 8.8% of the participants. Responses to the 3 subjective questions are shown in Table 3. The majority of the subjects (94.19%) noticed the need to replace their missing teeth with a statistically significant value (p value < 0.0001*). Most of the participants (66.7%) reported that losing teeth affected their appearance. Most of the participants (83.3%) reported that losing teeth affected their ability to chew food. The distribution of reasons that missing teeth were not replaced is shown in Fig. 4.

**Table 3:** Distribution table of missing teeth by level of education

	Missing anterior teeth		Missing posterior teeth	
Education Level	Number	Percentage	Number	Percentage
Primary	46	77.17%	46	67.66%
High school	26	17.93%	26	22.28%
Higher education	14	4.89%	14	10.05%

A total of 38.2% of participants’ income levels were between 1000 and 5000 SR, 39.2% were less than 1000 SR, and only 9.8% were above 10,000 SR. A total of 74.5% of the participants were married, and only 25.5% were single. A total of 52.9% of the participants had 1 missing anterior tooth or more, which dictates the need for esthetic restoration. A total of 58.8% of the participants had 3 missing posterior teeth or more, which dictates the need for functional restoration. There was a correlation between age and the number of missing teeth. Participants with an older age had a higher number of missing anterior and posterior teeth. Participants older than 45 years had the highest number of missing anterior and posterior teeth, with percentages of 72.28% and 60.05%, respectively (Fig. 4). In terms of the correlation between education level and the number of missing anterior and posterior teeth, the results show that participants with a higher level of education had a lower number of missing anterior and posterior teeth compared to participants with a lower level of education (Table 3). In terms of the relationship between education level and preferred treatment options, 24.51% of the participants with a secondary education level or higher selected a fixed treatment option (either implant 15.12% or fixed partial 13.96%) (Table 1).

There was a statistically significant difference between the level of education and the number of missing posterior teeth, with a p-value of 0.0055. Participants with a higher level of education had a lower number of missing posterior teeth.

Table 4 contains data on the patient-perceived and professionally assessed need for esthetic treatment categorized according to age group. Overestimation of esthetic treatment based on age group was highest in individuals above 45 years of age. Regarding the underestimation of esthetic treatment needs, the youngest age group consisting of individuals aged 18–25 years had the highest percentage. In Table 5, the overestimation of esthetic treatment needs based on education level fell in a narrow range and was lowest among the higher education level subjects. Underestimation of esthetic treatment needs covered a narrow range and was highest in the primary education level subjects. Differences in patient perceptions and professional assessments of the need for esthetic treatment were not significant across age and education groups.

**Table 4:** Two-by-two table of perceived esthetic needs and professionally assessed esthetic needs in different age groups

Age	Perceived esthetic need	Professionally assessed esthetic need		p value*	Overestimation %	Underestimation %
		Negative	Positive			
18–25	Yes	3	2	0.0833	50	100
	No	3	0			
26–35	Yes	6	4	0.1573	40	66.67
	No	9	2			
36–45	Yes	4	5	0.4142	44.44	71.43
	No	5	2			
> 45	Yes	9	20	0.6171	64.29	74.07
	No	5	7			

*For 2-by-2 tables, Bowker’s test is equivalent to McNemar’s test (P ≤ 0.05)

**Table 5:** Two-by-two table of perceived esthetic needs and professionally assessed esthetic needs by different educational levels

Education level	Perceived esthetic need	Professionally assessed esthetic need		p value*	Overestimation %	Underestimation %
		Negative	Positive			
Primary	Yes	12	17	0.1573	52.17	73.91
	No	11	6			
High school	Yes	7	10	0.2059	53.85	76.92
	No	6	3			
Higher education	Yes	3	4	0.6547	37.5	66.67
	No	5	2			

*For 2-by-2 tables, Bowker’s test is equivalent to McNemar’s test (P ≤ 0.05)

Table 6 shows the distribution of patient-perceived and professionally assessed needs for functional treatment among the different age groups. Underestimation of functional treatment needs covered a wide range and was highest in participants in the youngest age groups (18–35 years old). Overestimation of functional treatment needs was highest in participants in the oldest age group (> 45 years). The difference between patient-perceived and dentist-assessed needs was significant in the two age groups (26–35 and > 45). Table 7 shows the distribution of patient-perceived and professionally assessed needs for functional treatment based on education level. Overestimation of functional treatment need was lowest in subjects who had only higher education, while an underestimation of need was highest in participants in the same group. Differences between patient perceptions and professional assessments of the need for functional treatment were significant in participants in the primary and high school education level groups.

**Table 6:** Two-by-two table of perceived functional need and professionally assessed functional need categorized by age group

Age	Perceived functional need	Professionally assessed functional need		p value*	Overestimation %	Underestimation %
		Negative	Positive			
18–25	Yes	3	2	0.0833	50	100
	No	3	0			
26–35	Yes	6	11	0.0143*	60	100
	No	4	0			
36–45	Yes	5	7	0.5153	71.43	77.78
	No	2	2			
> 45	Yes	13	24	0.0290*	100	85.71
	No	0	4			

*For 2-by-2 tables, Bowker’s test is equivalent to McNemar’s test (P ≤ 0.05)

**Table 7:** Two-by-two table of perceived functional need and professionally assessed functional need categorized by educational level

Education level	Perceived functional need	Professionally assessed functional need		p value*	Overestimation %	Underestimation %
		Negative	Positive			
Primary	Yes	15	26	0.0116*	93.75	86.67
	No	1	4			
High school	Yes	9	13	0.0348*	81.82	86.67
	No	2	2			
Higher education	Yes	3	5	0.2615	33.33	100
	No	6	0			

*For 2-by-2 tables, Bowker’s test is equivalent to McNemar’s test (P ≤ 0.05)

## Discussion

Patient-centered prosthetic treatment planning has become a promising approach for replacing missing teeth to achieve patient satisfaction (Elias and Sheiham, 1998, Rosenoer and Sheiham, 1995). The current study was to determine factors that influenced patients’ perceptions of prosthetic treatment needs (both esthetic and functional), as well as to compare those perceptions to professionally assessed clinical needs.

Most of the study participants were above the age of 45 (47.67%) and had low educational levels (31.40%). This could be due to the inclusion and exclusion criteria, as any patient with no missing teeth was excluded from the study, and older people had more missing teeth. This outcome is inconsistent with a local study conducted in Qassim that found that most of the participants were aged above 50 years (30.8%) [17].

The main cause of tooth loss was dental caries in our study, which correlates with other studies [16, 18]. The reason for extraction is inaccurate because it relies on subjective information provided by individuals. Additionally, molars are considered the most commonly lost teeth and are mainly lost due to dental caries, rather than lower incisors, which are lost mainly due to reasons other than dental caries [19, 20].

According to the findings of our study, the number of missing teeth in participants increased with age. Older individuals may require more prosthetics due to their advanced age. Factors associated with aging, including as decreased salivary flow rate, quality, and quantity, decreased immunity, and the human body’s reduced ability to repair itself, could exacerbate the process of oral tissue degeneration [21].

Patients’ self-assessments of the impact of missing teeth on appearance varied among subjects. In our study, 66.7% of the subjects had aesthetic concerns about the loss of teeth. This is in agreement with earlier studies that reported wide variations in the impact of tooth loss [7, 10, 22]. Many of the participants (25.49%) had esthetic concerns about missing molars, while 11.76% of participants did not have concerns about losing premolars. The results obtained in our study were in agreement with a local study [12], which found that 14% of the subjects believed that their appearance was affected by missing molars, while 18% of subjects had no esthetic concerns about missing premolars. Osterberg et al. in 1984 [23] noted that esthetics is a priority for patients, followed by functional factors in replacing missing teeth.

A total of 2.9% of our study population preferred not to restore their missing teeth. In contrast to a Malaysian study reporting whether individuals lost anterior or posterior teeth, the subjects were not concerned about replacing those teeth [24]. Liedberg et al. in 1991 [25] found in a Swedish population that, though there was a high prevalence of list premolar and molar teeth, there was no desire to replace them. Gradual loss of teeth over time allows patients to adapt to their appearance and chewing ability, which is why the geriatric population may not perceive the need to replace missing teeth [26].

In line with the Akeel study, [12], some participants overestimated the treatment needs, and others did not state a need to replace several missing anterior and posterior teeth. Kayser et al. in 1988 [27] stated that the perceived need for replacing missing teeth was only noted if it affected appearance or mastication. On the other hand, old people with multiple missing teeth give priority to mastication rather than appearance [28]. The results from the present study revealed that there was disagreement between patients’ perceived need and professionally assessed needs in several patients; moreover, there was a significant difference between the need for functional restoration but not for esthetic restoration. This may reflect the existence of agreement between the dentist and patient about esthetic needs rather than functional needs, and perhaps the presence of visible spaces (Tables 4, 5, 6 and 7).

Patients’ perceived needs for replacement of missing teeth were affected by socioeconomic status. Individuals who had a lack of education, low financial resources and a need for preventive services were more likely to have neglected their dental health care [29]. The availability of governmental centers that provide free dental treatment made the major reason for not replacing missing teeth as the delayed appointments, which was 53.9%, followed by 33.3% of study subjects who gave an economic reason. These results could also explain the trend toward the most preferred treatment option, which is fixed partial dentures. Most recent studies were in agreement with our study (61.8%) of participants who knew about the treatment options from a dentist, which reflects the role of dentists in raising patient awareness about different options for missing teeth replacement [17, 30–32]. However, it is the dentist’s responsibility to spend time educating patients regarding available prosthetic options and clarifying the advantages and disadvantages of each option [30, 31].

One of the study’s limitations was its small sample size. Due to a lack of logistics and support, data were obtained using a random sampling technique. Because the current study used a questionnaire as its research tool, respondent bias may have contributed to the study’s limitations.

## Conclusions

Patients’ perceptions of functional and esthetic needs were variable among the population in comparison to professional assessment of patient’s needs. A higher level of education contributed to a lower number of missing posterior teeth. Overestimation of functional treatment need was lowest in subjects with higher education levels and highest in participants in the oldest age group (> 45 years). Dentists play a major role in raising a level of awareness about missing teeth replacement. The results of this study provides baseline data for future studies.

## Data Availability

The datasets used and/or analysed during the current study available from the corresponding author on reasonable request.
